# Is the visceral adiposity index a potential indicator for the risk of kidney stones?

**DOI:** 10.3389/fendo.2022.1065520

**Published:** 2022-12-01

**Authors:** Bingbing Hou, Xudong Shen, Qiushi He, Yang Chen, Yuexian Xu, Mingwei Chen, Junhua Xi, Zongyao Hao

**Affiliations:** ^1^ Department of Urology, the First Affiliated Hospital of Anhui Medical University, Hefei, China; ^2^ Institute of Urology, Anhui Medical University, Hefei, China; ^3^ Anhui Province Key Laboratory of Genitourinary Diseases, Anhui Medical University, Hefei, China; ^4^ Department of Endocrinology, the First Affiliated Hospital of Anhui Medical University, Hefei, China; ^5^ Department of Urology, the Second People’s Hospital of Hefei (Hefei Hospital Affiliated to Anhui Medical University), Hefei, China

**Keywords:** visceral adiposity index, kidney stones, risk factors, obesity, NHANES

## Abstract

**Objective:**

To determine whether the visceral adiposity index (VAI) was linked to the risk of kidney stones (KS) in the representative U.S. adults.

**Methods:**

We investigated 59842 participants who joined the 2007–2018 National Health and Nutrition Examination Survey. The association between the visceral adiposity index (VAI) and KS was identified by logistic regression analysis. Meanwhile, the subgroup analysis as well as the calculation of dose−response curves were also utilized to identify sensitive groups.

**Results:**

Data from 29384 participants were available, including 2781 self-reported ever experiencing KS diseases. Overall, the VAI was 0.74 (0.70, 0.78) in the KS group, while 0.55 (0.52, 0.57) in the control group. After adjusting for confounders, the prevalence of KS increased by 13% for each unit of VAI increment (OR = 1.13, 95% CI: 1.08, 1.19). Moreover, a linear relationship was found between the VAI and the prevalence of KS. By subgroup analysis, we found that a positive correlation between VAI and the risk of KS both in male (OR=1.14, 95%CI:1.07, 1.22) and female (OR=1.14, 95%CI:1.05, 1.24), White (OR=1.20, 95%CI:1.11, 1.28) and other race, all aged subgroups, nonhypertensive (OR=1.06, 95%CI:1.08, 1.25) and nondiabetic subgroups (OR=1.14, 95%CI:1.07, 1.21).

**Conclusions:**

Elevated VAI was strongly associated with KS in representative U.S. adults, which may be a promising indicator for the risk of kidney stones.

## Introduction

Kidney stones (KS) are widespread diseases of the urinary system, that clinically manifest as hematuria, renal colic, urinary tract infection, urinary tract obstruction, and, in severe cases may manifest as renal failure and are even life-threatening (1–3). Epidemiological studies show that KS diseases affect 1% to 20% of the population worldwide and the annual incidence of new cases is approximately 150-200 per 100,000 people, 25% of whom require hospitalization (2, 4, 5). Unfortunately, the prevalence of KS is on the rise worldwide and with a stone recurrence rate up to over 50% within five years (3, 6). To date, KS diseases have become a major public health problem, causing a serious economic burden to individuals and the country (7, 8). Therefore, how to prevent the occurrence of KS should be regarded as a high priority.

Currently, accumulated evidence suggests that metabolic syndrome (MS) is implicated in an elevated risk of KS (9–11), in which the role of obesity has attracted more attention from researchers (12, 13). Obesity has emerged as a public health problem in many countries worldwide (14, 15). A study conducted by Carbone et al. (16) showed that the prevalence of obesity in patients with KS was approximately 10-35%. Consistent with this, a higher rate of KS was also found in obese (11.2%) and overweight populations (9.1%) than in normal weight populations (6.1%) (17). Moreover, the risk of KS increased markedly with increasing body mass index (BMI) (18, 19). Although obesity was strongly associated with KS, reliable indicators of obesity to predict the risk of KS presence are extremely lacking.

Interestingly, researchers have recognized that adipose tissue has complex functions and that not all adipose tissue is harmful to the body, such as brown fat (20), so that high body weight and overall excess adiposity cannot simply be assumed to be poor health states. Therefore, defining and quantifying lipid accumulation in specific settings that may represent certain physiological risks can contribute to a deeper understanding of the role of adipose tissue in the pathophysiological processes of disease and its value in predicting the risk of disease occurrence. Several studies have shown that visceral adipose tissue is more strongly associated with diabetes, hypertension, cardiovascular disease and cardiometabolic risk factors than subcutaneous adipose tissue (21–25). However, traditional indicators of obesity, such as waist circumference (WC), BMI, waist-to-hip ratio, and waist-to-height ratio, did not differentiate visceral fat from subcutaneous fat. Although CT or MRI is considered the gold standard for the quantification of abdominal obesity, its expensive, complex and radiation-risky nature has prevented its large-scale implementation.

The visceral adiposity index (VAI) is an indicator of abdominal fat distribution and adipose tissue function proposed by Amato et al. (26). Its calculation includes not only anthropometric characteristics such as WC and BMI but also two metabolic indicators, triglycerides (TGs) and high-density lipoprotein cholesterol (HDL-C). These metrics and metabolism are strongly associated with the development of KS (18, 27) which allows us to more accurately assess the relationship between obesity and KS. Thus, we propose the hypothesis that VAI is associated with the risk of kidney stones. To explore this hypothesis, we investigated the link between VAI and KS diseases based on data from 29384 individuals.

## Materials and methods

### Study design and participants

**Figure 1:**
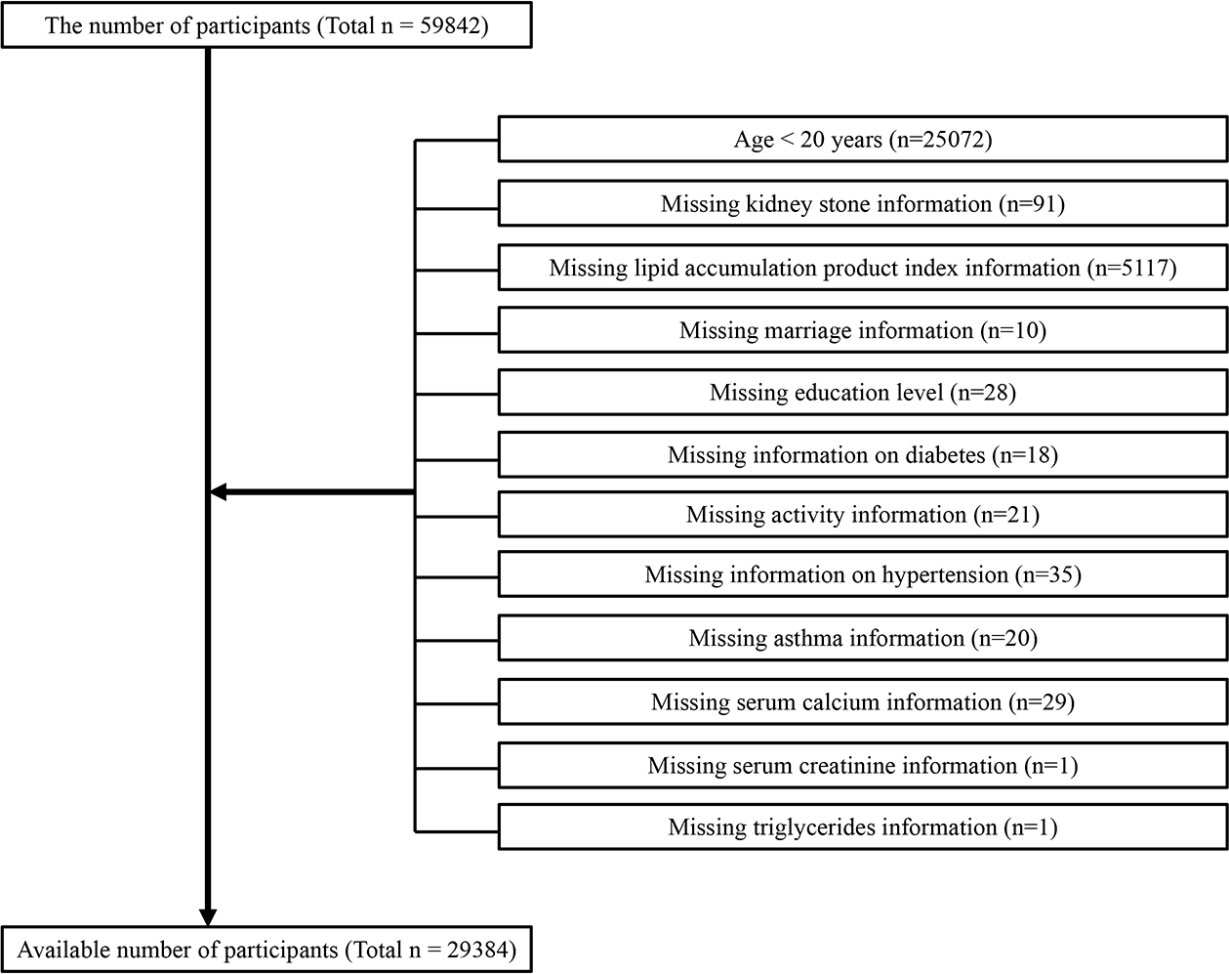
Flow chart of the participant selection process.

The baseline clinical data included in this analysis were derived from the National Health and Nutrition Examination Survey (NHANES) from 2007 to 2018, in which only participants aged over 20 years participated in the KS questionnaire; we preserved information on people who answered affirmatively as to whether they had kidney stones. A total of 59842 participated in the questionnaire. After excluding 30098 nontargeted participants, 29384 participants were available in this work (**Figure 1**), including 2781 self-reported ever experiencing KS diseases.

### Collection and definition of data

The VAI was developed as an exposure variable utilizing the below gender-specific equations. For males VAI=[WC(cm)/(39.68 + 1.88×BMI(kg/m2))]×(TG(mmol/L)/1.03)×(1.31/HDL(mmol/L)); for females VAI=[WC(cm)/(36.58 + 1.89×BMI(kg/m2))]×(TG(mmol/L)/0.81)×(1.52/HDL(mmol/L)) (28). Serum triglyceride concentrations were determined by Roche Cobas 6000 chemistry analysers. Questionnaires were used to assess the age at the time of surgery for the presence of KS, was the response to the questions of “Ever been told you have kidney stones?”. A variable designed to measure outcome was kidney stone occurrence.

In multivariate adjusted models, we summarized covariates that may confound the relationship between VAI and KS. The following factors were included in the study’s covariates: gender, race, age, education level, cholesterol concentration, marital status, alcohol intake, poverty-to-income ratio (PIR), smoking status, physical activity, diabetes, hypertension, and dietary intake factors. The 24-hour dietary recall was conducted on each participant in 2007 and 2018, and we will analyse the average consumption of both recalls. Detailed measurement protocols using the research variables are available at http://www.cdc.gov/nchs/nhanes/. All NHANES protocols were implemented following the Human Research Subject Protection Policy of the U.S and reviewed and standardized by the NCHS Research Ethics Review Committee. Informed consent forms were signed by all participants. There was no additional authorization or ethical review required for the release of NHANES data for this study.

### Statistical methods

The NHANES sample weights, clustering and stratification were used in all statistical analyses to illustrate the elaborate sampling methodology used to select a representative sample of U.S. noninstitutionalized adults. Continuous variables are presented as weighted survey means and 95% CIs, while categorical variables are presented as weighted surveys and 95% CIs. As VAI have skewed distributions, LN transformations convert them into normal distributions. In our study, we performed VIF covariate screening and removed covariates if the VIF value was greater than 5. Based on the guidelines (29), a multiple logistic regression model was conducted to explore the VAI, different VAI tertile groups and the prevalence of KS in three different models. Covariates were not adjusted in model 1, while model 2 adjusted for gender, race, age and education level as well as matrimony status, model 3 adjusted for covariates in Model 2 + hypertension, diabetes, alcohol use, PIR, smoking, asthma, physical activity, total kcal, total water, total sugar, serum calcium, serum cholesterol and serum creatinine. The relationship between the VAI and KS was further assessed using generalized additive model regression as well as smoothed curve fitting. Whenever a nonlinear relationship was identified, the inflection points were calculated by likelihood ratio tests. Multiple regression analysis was next performed on a stratified basis by gender, race, age, diabetes mellitus and hypertension. In addition, a log-likelihood ratio was utilized to test for heterogeneity in subgroup associations. Statistical significance was defined as P< 0.05. Empower software^®^ 4.0.2 was utilized for all analyses (www.empowerstats.com; X&Y Solutions, Inc., Boston, MA).

## Results

### Participant characteristics

The baseline demographic characteristics among the enrolled participants are summarized in **Table 1**. The VAI was 0.74 (0.70,0.78) in the kidney stones group, which was higher than that of 0.55 (0.52, 0.57) in the control group (P < 0.0001).

**Table 1:** Baseline characteristics of participants.

Characteristic	Nonstone formers (n=26603)	Stone formers (n=2781)	*P* value
Age(years)	46.60 (46.13, 47.06)	53.20 (52.57, 53.84)	<0.0001
Serum Cholesterol (mg/dl)	194.07 (193.08, 195.05)	192.28 (189.78, 194.79)	0.1434
Serum Calcium(mg/dl)	9.39 (9.38, 9.41)	9.37 (9.34, 9.40)	0.0587
Serum Creatinine(mg/dl)	0.87 (0.87, 0.88)	0.93 (0.91, 0.94)	<0.0001
VAI	0.55 (0.52, 0.57)	0.74 (0.70, 0.78)	<0.0001
Gender (%)			<0.0001
Male	47.78 (47.09, 48.47)	55.42 (52.73, 58.07)	
Female	52.22 (51.53, 52.91)	44.58 (41.93, 47.27)	
Race (%)			<0.0001
Mexican American	14.97 (13.08, 17.08)	11.35 (9.38, 13.66)	
White	65.69 (62.82, 68.45)	76.90 (73.77, 79.76)	
Black	11.13 (9.76, 12.67)	5.64 (4.65, 6.81)	
Other Race	8.21 (7.36, 9.15)	6.11 (4.91, 7.58)	
Education level (%)			0.0924
Less than high school	20.41 (19.00, 21.90)	20.08 (18.16, 22.15)	
High school	28.68 (27.50, 29.89)	31.34 (28.56, 34.27)	
More than high school	50.91 (49.04, 52.78)	48.57 (45.44, 51.72)	
Marital Status (%)			<0.0001
Cohabitation	63.36 (62.07, 64.63)	69.24 (66.67, 71.69)	
Solitude	36.64 (35.37, 37.93)	30.76 (28.31, 33.33)	
Alcohol (%)			0.7228
Yes	61.18 (59.71, 62.63)	59.99 (56.91, 62.99)	
No	18.51 (17.46, 19.61)	19.24 (17.05, 21.65)	
Unclear	20.31 (19.21, 21.46)	20.77 (18.05, 23.77)	
High Blood Pressure (%)			<0.0001
Yes	29.75 (28.75, 30.76)	46.35 (43.46, 49.27)	
No	70.25 (69.24, 71.25)	53.65 (50.73, 56.54)	
Diabetes (%)			<0.0001
Yes	8.54 (8.06, 9.05)	17.61 (15.87, 19.50)	
No	91.46 (90.95, 91.94)	82.39 (80.50, 84.13)	
Smoked (%)			<0.0001
Yes	43.65 (42.43, 44.87)	49.51 (46.67, 52.35)	
No	56.35 (55.13, 57.57)	50.49 (47.65, 53.33)	
Physical Activity (%)			0.0044
Never	26.05 (25.07, 27.07)	29.86 (27.65, 32.17)	
Moderate	31.94 (30.96, 32.95)	31.36 (29.06, 33.77)	
Vigorous	42.00 (40.88, 43.14)	38.77 (36.06, 41.56)	
Asthma (%)			0.0043
No	85.49 (84.77, 86.18)	82.72 (80.74, 84.54)	
Yes	14.51 (13.82,15.23)	17.28 (15.46,19.26)	
PIR			0.1303
<1.3	20.13 (18.90,21.43)	18.19 (16.46,20.07)	
≥1.3<3.5	32.50 (31.25,33.77)	35.03 (32.46,37.69)	
≥3.5	40.03 (38.15,41.93)	39.83 (36.56,43.18)	
3	7.34 (6.71,8.03)	6.95 (5.71,8.43)	
Total Kcal (%)			0.2454
Lower	39.08 (38.26,39.91)	40.26 (38.04,42.52)	
Higher	46.04 (45.04,47.04)	46.39 (43.69,49.12)	
Unclear	14.88 (14.09,15.72)	13.35 (11.62,15.29)	
Total Sugar (%)			0.9933
Lower	36.48 (35.63,37.35)	36.65 (33.86,39.54)	
Higher	37.22 (36.27,38.18)	37.11 (34.17,40.14)	
Unclear	26.30 (25.49,27.13)	26.24 (23.95,28.66)	
Total Water (%)			0.0507
Lower	38.88 (37.97,39.81)	37.34 (34.87,39.87)	
Higher	46.23 (45.28,47.18)	49.32 (46.55,52.09)	
Unclear	14.88 (14.09,15.72)	13.35 (11.62,15.29)	
Total Fat (%)			0.0507
Lower	38.88 (37.97,39.81)	37.34 (34.87,39.87)	
Higher	46.23 (45.28,47.18)	49.32 (46.55,52.09)	
Unclear	14.88 (14.09,15.72)	13.35 (11.62,15.29)	

Data of continuous variables are shown as the survey-weighted mean (95% CI), and the P value was calculated by survey-weighted linear regression. Data of categorical variables are shown as survey-weighted percentage (95% CI), P value was calculated by survey-weighted Chi-square test.

### VAI in participants with KS

The VIF for all indices of covariate screening was less than 5, and all variables were analyzed in the final adjusted model. The results showed that a positive correlation was identified between the VAI and the prevalence of KS. This positive correlation remained stable in the model 3 (OR=1.13, 95%CI:1.08, 1.19), indicating that the prevalence of KS increased by 13% with each unit increase in the LN-transformed VAI. Meanwhile, we converted the VAI to a categorical variable (triple quantile) from a continuous variable for sensitivity analysis and found that a significant 0.25-fold increase in KS presence was observed in Tertile 3 (OR= 1.25, 1.13, 1.39) compared to the lowest VAI lowest tertile (Tertile 1), as shown in **Table 2**.

**Table 2:** Analysis between the VAI with KS prevalence.

Characteristic	Model 1 OR (95%CI)	Model 2 OR (95%CI)	Model 3 OR (95%CI)
VAI Index	1.29 (1.23, 1.36)	1.20 (1.14, 1.26)	1.13 (1.08, 1.19)
Categories			
Tertile 1	1	1	1
Tertile 2	1.34 (1.21, 1.48)	1.21 (1.09, 1.35)	1.15 (1.04, 1.28)
Tertile 3	1.64 (1.49, 1.81)	1.40 (1.26, 1.55)	1.25 (1.13, 1.39)

Model 1 was adjusted for no covariates;

Model 2 was adjusted for race, gender, age, marital status and education;

Model 3 was adjusted for covariates in Model 2 + hypertension, diabetes, alcohol use, PIR, smoking, asthma, physical activity, total kcal, total water, total sugar, serum calcium, serum cholesterol and serum creatinine.

### Subgroup analysis

To assess the robustness of the VAI-KS relationship, subgroup analyses were conducted (**Table 3**). We found that VAI was positively correlated with prevalence of KS both in male (OR=1.14, 95%CI:1.07, 1.22) and female (OR=1.14, 95%CI:1.05, 1.24), White (OR=1.20, 95%CI:1.11, 1.28) and other race (OR=1.26, 95%CI:1.07, 1.49), age ≤ 40 years (OR=1.21, 95% CI:1.09, 1.34), >40 ≤ 60 years (OR=1.11, 95% CI:1.02, 1.21) and >60 years groups (OR=1.09, 95% CI:1.00, 1.18), nonhypertensive (OR = 1.06, 95% CI: 1.08, 1.25) and nondiabetic subgroups (OR=1.14,95%CI:1.07,1.21) We also controlled for interactions between BMI, age, gender, diabetes and hypertension. The results showed that age and race have an interaction effect on the association between VAI and KS prevalence.

**Table 3:** Subgroup analysis between the VAI and KS prevalence.

Subgroups	Model 1OR (95%CI)	Model 2OR (95%CI)	Model 3OR (95%CI)	*P* value for interaction*
Gender				0.9047
Male	1.25 (1.18, 1.33)	1.19 (1.11, 1.26)	1.14 (1.07, 1.22)	
Female	1.36 (1.27, 1.47)	1.26 (1.17, 1.37)	1.14 (1.05, 1.24)	
Race				0.0048
Mexican American	1.07 (0.97, 1.18)	1.02 (0.92, 1.13)	0.97 (0.88, 1.08)	
White	1.31 (1.22, 1.40)	1.27 (1.19, 1.36)	1.20 (1.11, 1.28)	
Black	1.23 (1.07, 1.41)	1.17 (1.01, 1.35)	1.12 (0.96, 1.29)	
Other Race	1.42 (1.22, 1.66)	1.34 (1.15, 1.57)	1.26 (1.07, 1.49)	
Age (years)				0.0075
20-39	1.40 (1.27, 1.54)	1.31 (1.19, 1.45)	1.21 (1.09, 1.34)	
40-59	1.27 (1.17, 1.37)	1.21 (1.11, 1.31)	1.11 (1.02, 1.21)	
60-80	1.15 (1.06, 1.24)	1.15 (1.05, 1.24)	1.09 (1.00, 1.18)	
Hypertension				0.0558
YES	1.17 (1.09, 1.25)	1.11 (1.03, 1.20)	1.07 (1.00, 1.16)	
NO	1.29 (1.21, 1.38)	1.19 (1.11, 1.28)	1.16 (1.08, 1.25)	
Diabetes				0.1713
YES	1.13 (1.02, 1.26)	1.08 (0.96, 1.21)	1.08 (0.96, 1.22)	
NO	1.26 (1.19, 1.33)	1.18 (1.11, 1.24)	1.14 (1.07, 1.21)	

Model 1 was adjusted for no covariates;

Model 2 was adjusted for race, gender and age;

Model 3 was adjusted for all covariates except the effect modifier;

* means only in model 3.

### Dose−response and threshold effects analysis of the VAI on KS

**Figure 2:**
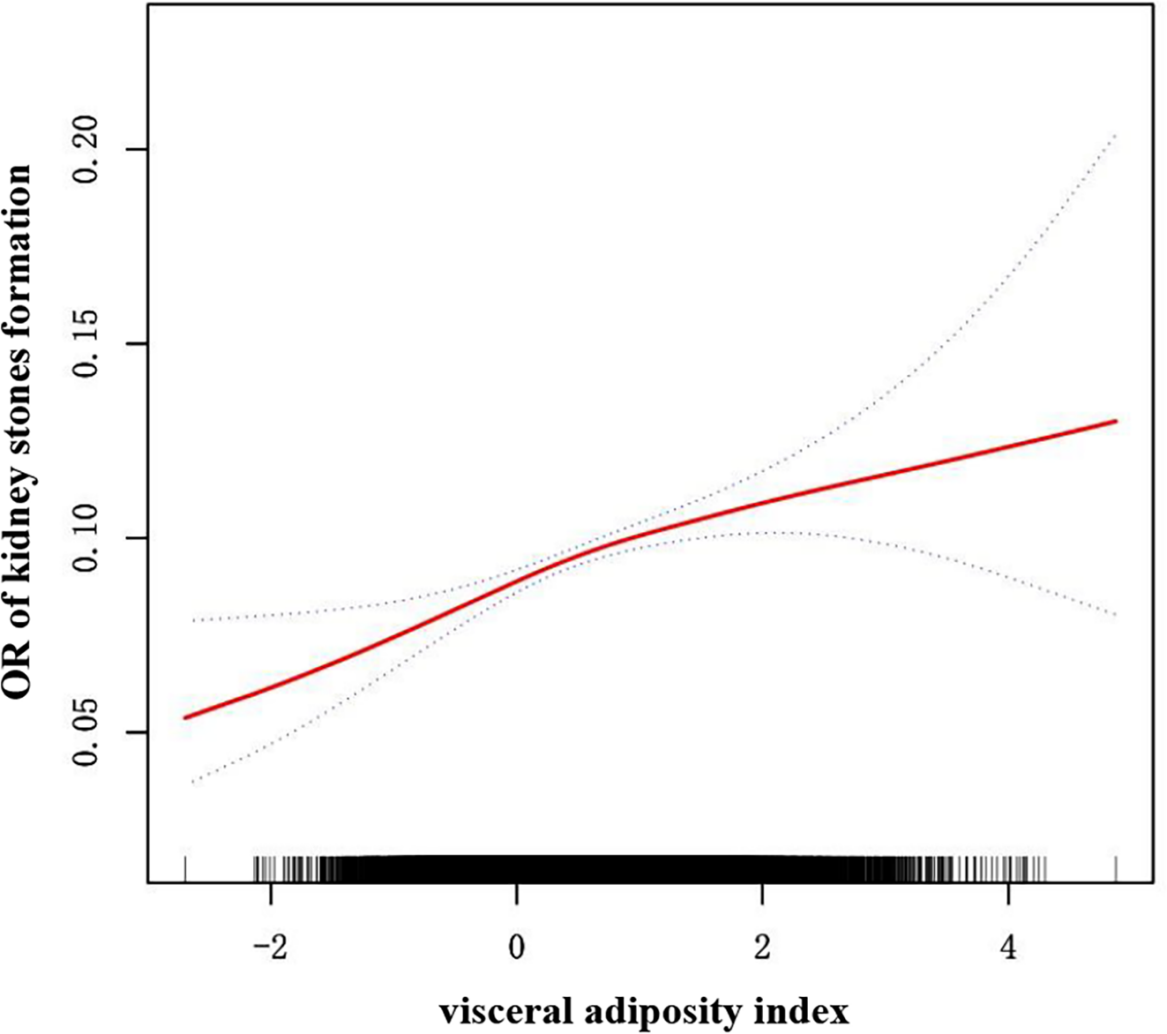
Density-dose-response correlation between the visceral adiposity index (VAI) and kidney stone formation. The area of the two blue dotted lines is indicated as the 95% CI. Each point illustrates the quantitative magnitude of the VAI index, which is linked into a continuous line. Adjustments were made for all individual covariates except effect modification factors.

Generalized additive model regression as well as smoothed curve fitting were applied to assess the relationship between the VAI and KS. Our results showed that VAI was linearly related to KS presence (**Figure 2**).

## Discussion

Although obesity is involved in the high prevalence of KS (11, 19, 27), reliable indicators of obesity to predict the risk of KS remain lacking. In this study, based on the NHANES database we demonstrated that a higher VAI was linked to a higher prevalence of KS in the representative U.S. adults after adjusting for confounders and the prevalence of KS increases by 13% for each unit of VAI increment after normalizing the VAI by LN transformation. Moreover, by converting the VAI from a continuous variable to a trichotomous categorical variable, sensitivity analysis showed that the VAI was reliable, with a significantly greater incidence of KS in the highest VAI group (Tertile 3). We also discovered that VAI was linearly related to KS presence in the dose response and threshold effect analyses. Our findings suggest that VAI may be a promising predictor of KS.

VAI as a specific index of visceral adiposity disorders has been widely associated with cardiovascular diseases, hypertension, human purine metabolism, insulin resistance and thyroid function (30–32). Recently, a retrospective study reported by Sönmez et al. (33) concluded that the VAI was positively correlated with the creatinine levels in patients with KS. However, evidence regarding the association between the VAI and KS are limited. In a retrospective study of 1,698 patients with stones, Trinchieri et al. (34) found that overweight and obese patients were commonly associated with increased excretion of urinary calcium, urinary oxalate and uric acid, which are strongly associated with the development of KS (3). Excessive visceral adipose tissue can lead to disorders of fatty acid metabolism and release free fatty acids, which induce inflammatory responses in a variety of cells such as macrophages and adipocytes (35, 36). Taguchi et al. (12) reported that mice treated with a high-fat diet exhibited increased accumulation of lipids in the kidney and triggered an inflammatory response, which ultimately led to the development of kidney stones. Although the above reports do not directly point to the relationship between VAI and KS, there may be a link between visceral adiposity and KS, which may be an idea to explain the better development of KS in patients with a high VAI.

KS is a common urological disease with a high global prevalence but an unknown mechanism. On the one hand, the specific mechanism of its occurrence is still unclear, and there is no effective etiologic prevention method; on the other hand, the treatment method of KS is constantly adjusted according to the progress of the patient’s stones, and early detection of KS can reduce the possibility of invasive operations for patients, reduce the pain suffered by patients and reduce the cost of treatment (37). Therefore, secondary prevention in the high-risk group is particularly important to achieve early detection, diagnosis, and treatment of KS. Our study showed that a high VAI is a statistically significant risk factor associated with KS. The VAI is related to the volume and area of visceral adipose tissue and is an index to evaluate the extent of visceral fat distribution and accumulation. Moreover, the VAI has higher sensitivity and specificity than traditional indicators such as waist circumference, BMI, and blood lipids (26). Our study supports that an elevated VAI was significantly associated with KS. Screening for VAI in high-risk groups provides new ideas for prevention and treatment of KS, which can prevent the occurrence of KS and provide early management of patients with KS.

Subgroup analysis showed that the relationship between the VAI and KS incidence was robust and generalized, and this positive correlation still existed in different gender, ethnicities, and age groups. This result needs to be supported by a larger sample size study. After further subgroup studies, the lowest VAI group (Tertile 1) showed a significant 25% increase in the prevalence of KS compared with the highest VAI group (Tertile 3), reflecting the considerable sensitivity of the VAI. A promising point in this study was that in different age subgroups, patients with a high VAI in the younger age group had a stronger correlation with KS presence than patients with a high VAI in the older age group, which gives us a new strategy for KS prevention, namely the management and control of the VAI in younger patients. We hypothesized that an elevated VAI is an important factor in the development of pro-KS and contributes to cardiovascular disease, metabolic syndrome and other disorders. However, the low age of the patient itself is a protective factor for cardiovascular disease, metabolic syndrome but has no significant protective effect on the development of KS, so the prediction of KS by the high VAI may have a higher specificity within younger individuals. No relevant reports in this regard have been published, and follow-up large-scale prospective studies are needed to confirm this causal relationship.

Our study has the following advantages. First, NHANES study participants are representative U.S. adults, which closely followed a well-designed study protocol with extensive guarantee and conformance. Second, we controlled for confounding variables and performed subgroup analyses to make our findings reliably and stably applicable to a wider range of individuals. Third, we further demonstrated that the VAI was linearly related to the prevalence of KS. However, the limitations of our study cannot be ignored. First, our cross-sectional investigation was based on the NHANES database, which prevented us from assessing a causal relationship between VAI and KS. Second, the prevalence of KS was obtained from patient self-reports, and recall bias was inevitable. Despite these limitations, our study clearly revealed a positive correlation between the VAI and KS presence.

## Conclusion

We demonstrated that the elevated VAI was strongly associated with KS in representative U.S. adults in a cross-sectional study. VAI may be a promising indicator for the risk of KS, which is beneficial for prevention guidance of KS.

## Data availability statement

The original contributions presented in the study are included in the article/supplementary material. Further inquiries can be directed to the corresponding authors.

## Ethics statement

The studies involving human participants were reviewed and approved by the NCHS Research Ethics Review Committee approved the NHANES survey protocol (https://www.cdc.gov/nchs/nhanes/irba98.htm). The patients/participants provided their written informed consent to participate in this study.

## Author contributions

ZH, MC and JX: Conceptualization, Methodology and Project administration; BH, QH and XS: Visualization, Investigation, Software and Writing - review & editing; YC, YX: Software and data collection. All authors commented on previous versions of the manuscript. All authors read and approved the final manuscript.

## Funding

This study was supported by the National Natural Science Foundation of China (82070724, 82000672) and the Natural Science Foundation of Anhui Province (1908085MH246, 2108085MH269).

## Acknowledgments

We thank all other researchers in our laboratory for their valuable help in our work.

## Conflict of interest

The authors declare that the research was conducted in the absence of any commercial or financial relationships that could be construed as a potential conflict of interest.

## Publisher’s note

All claims expressed in this article are solely those of the authors and do not necessarily represent those of their affiliated organizations, or those of the publisher, the editors and the reviewers. Any product that may be evaluated in this article, or claim that may be made by its manufacturer, is not guaranteed or endorsed by the publisher.
